# MUCOCUTANEOUS MANIFESTATIONS OF DENGUE FEVER

**DOI:** 10.4103/0019-5154.60359

**Published:** 2010

**Authors:** Emy Abi Thomas, Mary John, Bimal Kanish

**Affiliations:** *From the Department of Dermatology, Christian Medical College, Ludhiana, India.*; 1 *From the Department of Medicine, Christian Medical College, Ludhiana, India.*

**Keywords:** *Dengue fever*, *mucocutaneous*, *rash*

## Abstract

Dengue viral infection is a cause of considerable morbidity and mortality and may be associated with a variety of mucocutaneous manifestations that may provide important early clues to the diagnosis of this condition. Cutaneous and mucosal findings like confluent erythema, morbilliform eruptions, and hemorrhagic lesions may figure prominently in the clinical features of dengue. The differential diagnoses include a large number of bacterial and viral exanthems as well as drug rash.

## Introduction

Although it has been said that the eyes are the window to the soul, it may also be said that the skin is the window to within. The skin can provide important clues to systemic diseases, enabling the practitioner to make a tremendous contribution to patient care if cutaneous manifestations in a systemic disorder can be identified. This article focuses on the various mucocutaneous manifestations associated with dengue viral infection.

Dengue fever (DF) is a severe, flu-like illness that affects infants, children, adolescents, and adults. The incubation period of DF after the mosquito bite is between 3 and 8 days. The clinical features vary according to the age of the patient. Infants and young children usually have only a nonspecific febrile illness, with a rash that is hard to distinguish from other viral illnesses.[1] The more severe cases usually occur in older children and adults and are characterized by a rapidly rising temperature (> 39°C) that lasts approximately 5 to 6 days and sometimes may be biphasic. During the febrile period, the patient may experience severe headache, retro-orbital pain, myalgia, arthralgia, nausea, and/or vomiting. More than 50% of infected patients report having a rash during this period that initially is macular or maculopapular and becomes diffusely erythematous.[2] Minor hemorrhagic manifestations such as petechiae, epistaxis, and gingival bleeding occur in some patients.

## Etiopathogenesis

There are four serotypes of the dengue virus (DEN 1-4). Dengue virus is a single-stranded RNA virus transmitted mainly by the mosquito *Aedes aegypti*. Various hypotheses regarding the etiology are as follows:

1. Viral replication, which occurs primarily in macrophages, although dendritic cells (Langerhans cells) in skin may be the early targets of infection.[3]
2. Direct infection of the skin by dengue virus.[4]
3. Immunologic and chemically mediated mechanisms induced by interaction of the virus with the host.[4]

However, De Andino *et al*. concluded that the absence of evidence for direct viral involvement or for immune complexes in the skin lesions could be due to viral host interaction inducing release of unidentified chemical mediators in the skin and that the rash has nothing to do with the direct viral invasion or with the presence of immune complexes.[5]

Infections with the dengue virus can cause a spectrum of three clinical syndromes with classic DF, dengue hemorrhagic fever (DHF), and dengue shock syndrome (DSS). World Health Organization criteria exist for the classification of dengue into these three clinical categories [Table 1];[6] however, there is a significant overlap between the categories.[7]

**Table 1:** World Health Organization criteria for dengue fever, dengue hemorrhagic fever, and dengue shock syndrome

Dengue fever	Dengue hemorrhagic fever	Dengue shock syndrome
An acute febrile illness with more than two of the following manifestations:	All of the following must be present	All four criteria for DHF must be met plus evidence of circulatory failure manifested by rapid and weak pulse and narrow pulse pressure or hypotension for age, systemic pressure <80 mmHg for those < age 5 years or <90 mmHg for those > age 5 years
	Fever or history of acute fever lasting 2-7 days		
Headache	Bleeding, evidenced by at least one of the following:		
Retroorbital pain			
Myalgia	Positive tourniquet test result		
Arthralgia	Petechiae, ecchymoses or purpura	
Rash	Bleeding from the mucosa, GI tract, infection sites, or other location.	
Hemorrhagic manifestation		Cold clammy skin and restlessness
Leukopenia	Hematemesis or melena	
AND	Thrombocytopenia (<1,00,000 cells/mm^3^)	
Supportive serology	Evidence of plasma leakage caused by increased vascular permeability, manifested by at least one of the following:	
OR		
Occurrence at the same location and time as other confirmed cases of DF	Increase in hematocrit ≥20% above average for age, sex, and population	
Laboratory criteria		
Isolation of dengue virus ≥ fourfold change in antibody titers	Decrease in hematocrit after volume replacement treatment ≥20% of baseline.	
Demonstration of dengue virus antigen	Signs of plasma leakage such as pleural effusion, ascites and hypoproteinemia	
Detection of dengue virus genomic sequence		

## Cutaneous Manifestations

**Figure 1:**
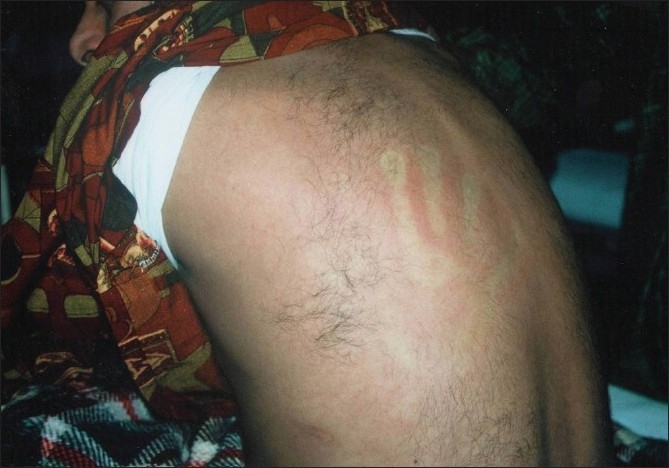
Diffuse exanthematous rash

**Figure 2:**
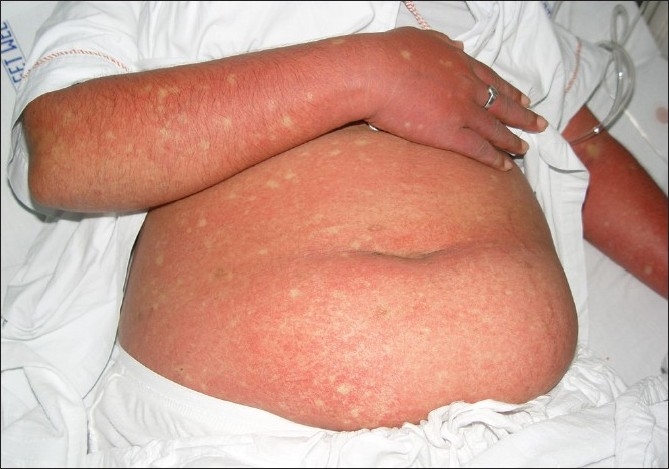
Confluent erythematosus rash with island of sparing

**Figure 3:**
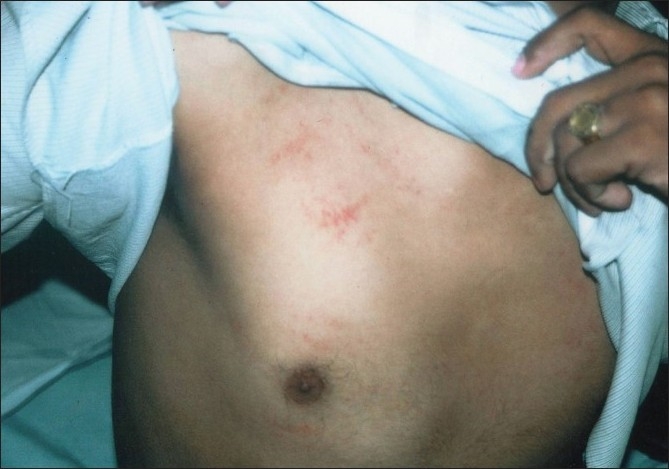
Petechial rash over chest

The characteristic exanthem of DF is estimated to occur in 50-82% of patients with DF.[8,9] Cutaneous findings figure prominently in the clinical manifestations of DF and DHF. In DF, the initial rash is a transient flushing erythema of face that typically occurs shortly before or within the first 24-48 hours of the onset of symptoms and is thought to be the result of capillary dilatation. The second rash usually occurs 3-6 days after the onset of fever and it is characterized by asymptomatic maculopapular or morbilliform eruption [Figure 1]. In some cases, individual lesions may coalesce and are then seen as generalized confluent erythema with petechiae and rounded islands of sparing-“white islands in a sea of red”[8,10] [Figure 2] and is thought to be due to an immune response to the virus. The rash in DF is usually asymptomatic, pruritus being reported in a substantial minority of patients in different studies; that is, 16% and 27.6%.[11,12] Some patients display only the initial rash and recover completely, while others develop the more generalized eruption.[13] The generalized rash characteristically starts on the dorsum of the hands and feet and spreads to the arms, legs, and torso and it lasts for several days and subsides without desquamation. The morbilliform, maculopapular rash usually spares palms and soles. Less frequently, rashes of two other types may occur.[11] An eruption of fine macule over pressure areas may accompany the premonitory symptoms and herald the onset of fever. In some cases, the end of the fever is also marked by cutaneous changes in the form of a purpuric eruption on the hands, forearms, feet and legs, and in the mouth. Hemorrhagic manifestations on the skin such as petechiae [Figure 3], purpura, or ecchymosis with positive tourniquet test are commonly seen in DHF and DSS and rarely in DF. Tourniquet test is performed by inflating a blood pressure cuff on the upper aspect of arm to a point midway between systolic and diastolic pressures for 5 minutes. The test is considered positive when >20 petechiae/2.5 cm^2^ are observed.[13] Hemorrhagic manifestations usually appear 4-5 days after the onset of fever.

## Mucosal Manifestations

**Figure 4:**
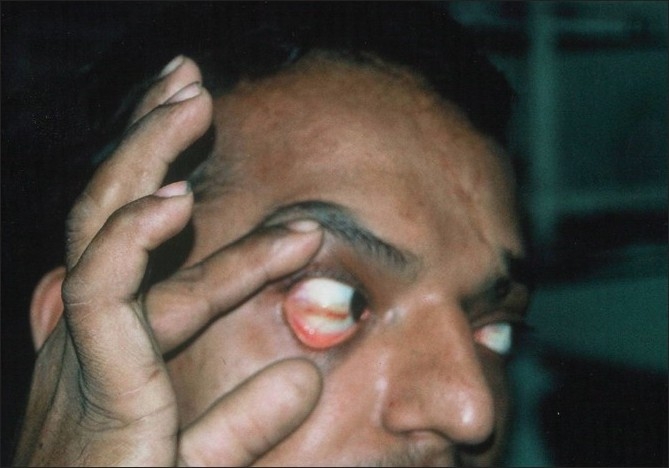
Scleral injection

Mucosal involvement is estimated to occur in 15% to 30% of patients with dengue viral infections and more commonly in patients with DHF than with DF.[12] The mucosal manifestations noted in dengue viral infections are conjunctival and scleral injection [Figure 4], small vesicles on the soft palate, erythema and crusting of lips and tongue. Chadwick *et al*.[11] reported conjunctival involvement in 14% of patients; however, some reports have shown a higher percentage of mucosal involvement, e.g. scleral injection (90%) and vesicles on the soft palate (> 50%).[14]

## Histopathology

In some of the studies on dengue skin pathology, biopsies performed on the local skin lesions showed that the epithelium was not involved and no inclusion bodies were found. The chief abnormality was found in and about the small blood vessels and consisted of endothelial swellings, a site of flavivirus replication, perivascular edema, and infiltration with mononuclear cells.[5,15] The epidermis and the rest of the dermis and subcutaneous tissues were normal.

## Immunofluorescence

In the study by De Andino *et al*.,[5] direct immunofluorescence of involved skin was negative for the deposition of immunoglobulins and complement and, for the presence of dengue viral antigen. Also attempts to isolate dengue virus from the involved skin were negative in all cases even though the virus was isolated from the serum of two of these patients.

In contrast to the above findings, in an immunofluorescence study of skin rash in DHF patients in Thailand, deposits of dengue antigen, IgM, and complements were found in the upper dermal plexus of 6/53 patients.[16]

The skin eruption with the accompanying systemic signs and symptoms may mimic other viral and nonviral diseases, making laboratory confirmation necessary for a correct diagnosis. The incidence of rash may vary with the virus strain.[17]

## Differential Diagnosis

When confronted with a febrile patient who has a rash similar to that seen with DF, the differential diagnosis is quite broad. The initial flushing erythema of the chest, head, and neck in association with fever can be seen in the early stages of many viral and bacterial infections [Table 2]. The generalized morbilliform eruption in association with fever can be seen in the later stages of various viral exanthems and bacterial infections [Table 3].

**Table 2:** Differential diagnosis of classic dengue fever associated flushing erythema

Disease	Incubation/presenting symptoms	Morphology	Distribution of rash	Associated findings	Laboratory finding
Chikungunya fever	3-12 days, fever, arthralgia, myalgia, headache ± vomiting	Flushing erythema	Face, upper chest	Severe joint pains, conjunctival injection	Normal CBC, raised ESR, raised C-reactive protein, Chikungunya IgM positive
Scarlet fever	2-4 days, fever, pharyngitis ± vomiting, abdominal pain, ± convulsions	Pinpoint papules on an erythematous back ground (sand paper), linear petechiae (pastia's lines), membranous desquamation of palms/soles	Generalized, spares palms and soles	Exudative pharyngitis	ASO titer positive, leucocytosis, raised ESR
Kawasaki disease	High fever, irritability	Flushing macular erythema, non pruritic erythematous plaques, erythema marginatum, desquamation of palms/soles	Most prominent on trunk and extremities	Conjunctivitis, strawberry tongue, coronary artery aneurysms, lymphadenopathy	Leucocytosis, raised ESR, thrombocytosis during second to third week, sterile pyuria in the 1^st^ wk
Toxic shock syndrome	Sudden onset fever	Erythroderma or scarlatiniform rash	Generalized	Hypotension, renal involvement, focus of infection	Raised serum creatinine phosphokinase level
Erythema infectiosum (fifth disease Parvovirus B-19)	6 days, nonspecific fever and malaise	Macular erythema on face (1-4 days)	Slapped cheek appearance of face followed by extremities, lacy rash over extensor surfaces	Aplastic crisis in sickle cell disease, women may develop arthritis, spontaneous abortions	IgM anti-bodies positive

**Table 3:** Differential diagnosis of classic dengue fever associated morbilliform eruption

Disease	Incubation/presenting symptoms	Morphology	Distribution of rash	Associated findings	Laboratory findings
Measles	8-12 days, rhinitis, cough, fever	Erythematous macules and papules, later becomes confluent	Begins on neck and face, spreads down and become generalized and as it fades it leaves a brownish hue with fine desquamation	Koplik's spots, exudative conjunctivitis, photophobia, pneumonia	Leucopenia, low ESR, IgM positive (measles, immunoglobulin)
German measles (Rubella)	14-23 days, mild URI, fever, eye pain, lymphadenopathy	Pinpoint maculopapular rash	Begins on face and spreads to trunk and extremities	Tender cervical lymphadenopathy, transient polyarthralgia and polyarthritis irritability, febrile seizures	Nasal culture for virus antibody titers
Roseola (Exanthem subitum HHV-6)	5-15 days, high fever for 3-5 days, diarrhea, cough	Pale pink macules and papules	Trunk, neck and proximal extremities		Leucocytosis at the onset and leucopenia later
Infectious mono-nucleosis	Fever, malaise	Polymorphic macularerythema ± petechiae, urticaria, erythema multiforme like lesions	Generalized	Pharyngitis, lymphadenopathy, pinhead petechiae at the junction of soft and hard palate (Forschheimer's spots)	Leukocytosis, atypical lymphocytes LFT (transaminases and bilirubin) may be elevated, serology for heterophilic antibodies positive
Secondary syphilis	Fever, headache, pharyngitis	Polymorphic macules/papules/psoriasiform papules	Generalized	Lymphadenopathy ± condyloma lata ± moth eaten alopecia	VDRL positive
Typhoid fever	Fever, vomiting, diarrhea, headache	2-3 mm pink grouped papules (Rose spots) generalized erythema “erythema typhosum”	Generalized, trunk		Rose spot cultures may be Salmonella typhi positive
Chikungunya fever	3-12 days, fever, arthralgia, myalgia, headache, vomiting	Flushing erythema maculopapular lesions, ± petechiae	Trunk and extremities	Severe joint pains, conjunctival injection	Normal CBC, raised ESR, raised C-reactive protein, Chikungunya IgM positive
Lepto-spirosis	Acute phase: Fever, headache, myalgia, pharyngitis	Morbilliform rash	Generalized	Immune phase: hemorrhage, jaundice, organ failure	Leukocytosis, microscopic agglutination test is positive
Acute retroviral syndrome (HIV)	Fever, fatigue headache	Maculopapular rash	Trunk and upper arms ± palms and soles	Myalgia, lymphadenopathy	HIV RNA, P24 antigen
Rocky mountain spotted fever	3-12 days, fever, malaise, headache	Erythematous macules, evolves to petechiae and purpura	Begins on wrist, ankles; spreads centrally; seen on palms and soles	Hepatosplenomegaly, hyponatremia, myalgias, CNS involve ment	Rickettsial group specific serologic tests positive
Drug exanthem	4-6 days, fever, malaise	Maculopapular or urticarial	Generalized symmetric often spares the face, palms and soles may be involved	Periorbital edema, fever	ESR is low

As illustrated in Tables 2 and 3, the differential diagnosis of DF is broad. It is imperative to exclude Chikungunya fever as its clinical presentation is almost indistinguishable from DF and a similar epidemic is occurring in various parts of the world.

Clinical features favoring Chikungunya fever over DF include a more rapid onset of symptoms, more severe rash, worse conjunctival injection, shorter febrile period,[18] and fewer signs of easy bleeding.[19] Although both conditions are associated with severe arthralgias, patients with Chikungunya fever are more likely to contort themselves into characteristic postures because of severe joint pain.[19]

The rash in scarlet fever begins 12-48 hours after the onset of fever, starts as erythema of the neck, chest, and axillae, and within 4-6 hours it becomes generalized. The rash consists of tiny papules on an erythematous background. Visually, it resembles ‘sunburn with goose pimples’ and feels like sand paper. Pastia's lines (linear petechial streaks) are seen in the axillary, antecubital, and inguinal areas. The cheeks are flushed with circumoral pallor. The tongue is initially white with bright red papillae, but later becomes beefy red (red strawberry tongue).[20] After 7-10 days, desquamation occurs, most severely affecting the hands and feet, and lasts for 2-6 weeks.

A skin eruption is seen in over 80% of patients with Kawasaki disease[21] and is usually morbilliform or macular but may also be scarlatiniform, erythema multiforme-like, or pustular. A characteristic cutaneous feature is erythema of the perineum, which often desquamates within 48 hours. The conjunctival injection of Kawasaki disease is characterized by perilimbal sparing and lack of increased tearing or exudates.

The cutaneous manifestations are more extensive and predictable in staphylococcal toxic shock syndrome than in streptococcal toxic shock syndrome. Patients usually develop a diffuse scarlatiniform exanthem that starts on the trunk and spreads centripetally with erythema and edema of palms and soles. Erythema of the mucous membranes, a strawberry tongue, and hyperemia of the conjunctivae are also present. Desquamation of the hands and feet occurs 1-3 weeks after the onset of symptoms.[22]

Erythema infectiosum, which is also known as fifth or slapped cheek disease, is another condition that has to be differentiated from DF, though it is most common in children between 4 and 10 years of age. The initial stage of the exanthem consists of bright red macular erythema of the cheeks, with sparing of the nasal bridge and circumoral regions. One to four days later, the second stage appears in the form of erythematous macules and papules, which progress to a lacy, reticulate pattern, occurring most often on the extremities and to a lesser extent on the trunk.[23]

Measles classically presents with a prodrome and a pathognomonic enanthem Koplik's spots, appears during the prodrome, and is composed of gray-white papules on the buccal mucosa opposite the premolar teeth.[24] The exanthem appears over 2-4 days and consists of erythematous macules and papules that begins on the forehead, hairline, and behind the ears and then spread in a cephalocaudal direction. On the fifth day, the exanthem starts to fade in the same order as it appeared.

Cutaneous manifestations of rubella typically presents 1-5 days following the prodrome as an eruption of erythematous macules and papules on the face and spreads in a cephalocaudal direction. In 2% of cases, petechiae on the soft palate occur late in the prodromal phase or early in the eruptive phase. The cutaneous eruption tends to fade in 2-3 days in the same order as it appeared.[25]

Roseola infantum is a viral disease common in infants, characterized by high fever and skin rash. Cutaneous eruption in roseola is erythematous almond-shaped macules and papules on the trunk, neck, and proximal extremities. An enanthem of red papules on the soft palate and uvula (Nagayama's spots) may be seen; HHV-6 infection should be suspected in infants with febrile convulsions, even those without the examthem.[26]

Cutaneous findings are observed in 5% of patients with infectious mononucleosis and include macular, papular, urticarial, petechial, scarlatiniform, or erythema multiforme like eruptions. Palatal petechiae may be present. Up to 90% of patients with infectious mononucleosis who receive ampicillin or amoxicillin develop a maculopapular eruption.[27,28]

In secondary syphilis, the most commonly observed clinical presentation is a generalized nonpruritic papulosquamous eruption. Snail track ulcers in the oral cavity and Condyloma lata of the moist areas are other features.[29]

In typhoid fever, the characteristic cutaneous sign is the ‘rose spot’. It is a 2-8 mm, pink, blanching papule that is usually found on the anterior trunk in groups of 5-15 lesions.[30] They occur in up to 50% of patients with typhoid fever and less often in nontyphoidal enteric fever. Rose spots often occur in crops during the second to fourth weeks of the illness, and salmonella can usually be cultured from these lesions.

In leptospirosis, skin manifestation is not very common. Sometimes a blotchy macular erythema or purpura may be seen on the legs.[31]

The earliest cutaneous manifestation of HIV infection may be an exanthem occurring as a manifestation of primary HIV infection. The generalized morbilliform exanthem of ‘acute retroviral syndrome’ typically spares the palms and soles and it usually lasts for 4-5 days.[32]

Rashes are among the most common adverse reactions to drugs. They occur in many forms and mimic many dermatoses. They occur in 2-3% of hospitalized patients.[33–35] Exanthematous or maculopapular drug eruption are the commonest and they occur suddenly often with fever, 7-10 days after the drug is first taken. They are generalized, symmetric, and often pruritic. Maculopapular eruptions are often indistinguishable from viral exanthems and it is usually due to ampicillin or amoxicillin, but any drug can trigger it. Red macules and papules become confluent in a symmetric, generalized distribution that often spares the face. Itching is common. Mucous membranes, palms, and soles may be involved. Fever may be present from the onset. These eruptions are identical in appearance to a viral exanthem and routine laboratory tests usually fail to differentiate the two diseases. Lesions clear rapidly following withdrawal of the implicated agent and may progress to a generalized exfoliative dermatitis if use of the drug is not discontinued.

DF should be considered in the differential diagnosis of fever and rash in a patient residing or returning from an endemic area,[13] and dermatologists should be aware of the distinctive exanthem of DF. Recognition of DF rash permits a rapid and early diagnosis, which is critical, as DF can progress to life-threatening DHF or DSS.

## References

[1] Ligon BL (2004). Dengue fever and Dengue Hemorrhagic fever: A review of history, transmission, treatment and prevention. Semin Pediatr Infect Dis.

[2] World Health Organization Dengue fever in Indonesia.

[3] Wu SJ, Grouard-Vogel G, Sun W, Mascola JR, Brachtel E, Putvatana R (2000). Human skin Langerhans cells are targets of dengue virus infection. Nat Med.

[4] Bhamarapravati N (1980). Pathology and pathogenesis of DHF.

[5] de Andino RM, Botet MV, Gubler DJ, García C, Laboy E, Espada F (1985). The absence of Dengue virus in the skin lesions of Dengue Fever. Int J Dermatol.

[6] World Health Organization (1997). Dengue hemorrhagic fever: Diagnosis, treatment, prevention and control.

[7] Bandyopadhyay S, Lum LC, Kroeger A (2006). Classifying dengue: A review of the difficulties in using the WHO case classification for dengue hemorrhagic fever. Trop Med Int Health.

[8] Waterman SH, Gubler DJ (1989). Dengue fever. Clin Dermatol.

[9] Itoda I, Masuda G, Suganuma A, Imamura A, Ajisawa A, Yamada K (2006). Clinical features of 62 imported cases of dengue fever in Japan. Am J Trop Med Hyg.

[10] Radakovic-Fijan S, Graninger W, Müller C, Hönigsmann H, Tanew A (2002). Dengue hemorrhagic fever in a British travel guide. J Am Acad Dermatol.

[11] Chadwick D, Arch B, Wilder-Smith A, Paton N (2006). Distinguishing dengue fever from other infections on the basis of simple clinical and laboratory features: Application of logistic regression analysis. J Clin Virol.

[12] Thomas EA, John M, Bhatia A (2007). Cutaneous manifestation of dengue viral infection in Punjab (North India). Int J Dermatol.

[13] Pincus LB, Grossman ME, Fox LP (2008). The exanthem of dengue fever: Clinical features of two US tourists traveling abroad. J Am Acad Dermatol.

[14] Sanford JP (1986). World Health Organization: Dengue haemorrhagic fever: Diagnosis, treatment and control. Harrison's Principles of Internal Medicine.

[15] Sabin AB (1952). Research on dengue during World War II. Am J Trop Med Hyg.

[16] Boonpucknavig S, Boonpucknavig V, Bhamarapravati N, Nimmannitya S (1979). Immunofluorescence study of skin rash in patients with dengue hemorrhagic fever. Arch Pathol Lab Med.

[17] Sabin AB, Rivers TM, Horsfall FL (1959). Viral and Rickettsial infections of man.

[18] Nimmannitya S, Halstead SB, Cohen SN, Margiotta MR (1969). Dengue and Chikungunya virus infection in a man in Thailand, 1962-1964: Observations on hospitalized patients with hemorrhagic fever. Am J Trop Med Hyg.

[19] Carey DE, Myers RM, DeRanitz CM, Jadhav M, Reuben R (1969). The 1964 Chikungunya epidemic at Vellore, South India, including Observations on concurrent Dengue. Trans R Soc Trop Med Hyg.

[20] Bialecki C, Feder HM, Grant-Kels JM (1989). The six classic childhood exanthems: a review and update. J Am Acad Dermatol.

[21] Burns JC (2001). Kawasaki disease. Adv Pediatr.

[22] James WD, Berger TG, Elston DM (2006). Andrew's diseases of the skin: Clinical Dermatology.

[23] Cherry JD (1999). Parvovirus infections in children and adults. Adv Pediatr.

[24] Koplik H (1896). The diagnosis of the invasion of measles from a study of the exanthema as it appears on the buccal mucous membranes. Arch Pediatr.

[25] Wesselhoeft C (1947). Rubella (German measles). N Engl J Med.

[26] Segondy M, Astruc J, Atoui N, Echenne B, Robert C, Agut H (1992). Herpes virus 6 infection in young children. N Engl J Med.

[27] Mccarthy JT, Hoagland RJ (1964). Cutaneous manifestations of infectious mononucleosis. JAMA.

[28] Contratto AW (1944). Infectious mononucleosis: A study of one hundred and ninety six cases. Arch Intern Med.

[29] Chapel TA (1980). The signs and symptoms of secondary syphilis. Sex Transm Dis.

[30] Marzano AV, Mercogliano M, Borghi A, Facchetti M, Caputo R (2003). Cutaneous infection caused by Salmonella typhi. J Eur Acad Dermatol Venereol.

[31] Karande S, Bhatt M, Kelkar A, Kulkarni M, De A, Varaiya A (2003). An observational study to detect leptospirosis in Mumbai, India, 2000. Arch Dis Child.

[32] Tindall B, Barker S, Donovan B, Barnes T, Roberts J, Kronenberg C (1988). Characterization of the acute clinical illness associated with human immunodeficiency virus infection. Arch Intern med.

[33] Bigby M, Jick S, Jick H, Arndt K (1986). Drug induced cutaneous reactions: A report from the Boston collaborative drug surveillance program on 15,438 consecutive in patients, 1975-1982. JAMA.

[34] Roujeau JC, Stern RS (1994). Severe adverse cutaneous reactions to drugs. N Engl J Med.

[35] Alanko K, Stubb S, Kauppinen K (1989). Cutaneous drug reactions-clinical types and causative agents: a five year survey of in-patients (1981-1985). Acta Derm Venereol.

